# Prognostic value of myositis-specific antibodies in patients with idiopathic interstitial pneumonia

**DOI:** 10.1186/s12890-024-03326-w

**Published:** 2024-10-10

**Authors:** Hiroki Wakabayashi, Kotaro Iwasaki, Yu Murakami, Kenta Takashima, Kaichi Kaneko, Yasuo Matsuzawa

**Affiliations:** 1 Division of Respiratory Medicine, Department of Internal Medicine, Toho University Sakura Medical Center, 564-1 Shimoshizu, Sakura, Chiba 285-8741 Japan; 2 Division of Rheumatology, Department of Internal Medicine, Toho University Sakura Medical Center, Sakura, Japan

**Keywords:** Idiopathic interstitial pneumonia, Interstitial lung disease, Myositis, Antisynthetase antibodies, Prognostic factors, anti-PL-7 antibody, anti-PL-12 antibody

## Abstract

**Background:**

Patients with idiopathic interstitial pneumonia (IIP) often exhibit positivity for myositis-specific antibodies (MSA). However, the significance of this finding remains unclear. In this study, we investigated the association of MSA with the prognosis and risk of acute exacerbation in patients with IIP.

**Methods:**

We retrospectively reviewed the medical records of patients with IIP and examined the effect of each MSA subtype on survival and acute exacerbation.

**Results:**

Of 240 patients with IIP, 48 (20%) exhibited positivity for MSA. The MSA subtypes included: PL-7 (antithreonyl; *n* = 16, 6.7%); signal recognition particle (*n* = 13, 5.4%); PL-12 (antialanyl; *n* = 9, 3.8%); Mi-2 (*n* = 8, 3.3%); OJ (anti-isoleucyl; *n* = 7, 2.9%). During the 382 days (382 ± 281 days) of observation, 32 (13%) patients expired, and 27 (11%) experienced an acute exacerbation. Cox proportional hazards regression analysis demonstrated that age at the initial visit (hazard ratio [HR]: 1.072; 95% confidence interval [CI]: 1.017–1.131; *P* = 0.01), PL-7 (HR: 4.785; 95% CI: 1.528–14.925; *P* = 0.007), and PL-12 (HR: 3.922; 95% CI: 1.198–12.82; *P* = 0.024) were independent predictors of survival time. PL-7 (HR: 3.268; 95% CI: 1.064–10; *P* = 0.039) and PL-12 (HR: 5.747; 95% CI: 1.894–7.544; *P* = 0.002) were independent predictors of time from first visit to acute exacerbation.

**Conclusion:**

Detecting MSA in patients with interstitial lung disease may be useful in predicting prognosis and providing a rationale for intensive treatment.

## Background

Myositis-specific antibodies (MSA) are useful autoantibodies in diagnosing idiopathic inflammatory myopathies (IIM). This group of diseases is characterized by progressive muscle weakness and infiltration of inflammatory cells into skeletal muscles, with an autoimmune component [1]. Patients with IIM often exhibit positivity for MSA with a specificity > 90% [1]. MSA can be detected using immunoblotting, simplifying their identification in routine clinical practice. It has been reported that MSA is associated with interstitial lung disease (ILD). The most common anti-aminoacyl tRNA synthetase (anti-ARS) antibodies are present in patients with anti-ARS syndrome; its characteristic presentation includes ILD, skin rash, arthritis, and Raynaud’s phenomenon [1]. Screening for MSA in patients with idiopathic interstitial pneumonia (IIP) is useful for diagnosing IIM and other autoimmune diseases [2, 3].

IIP is ILD for which a specific cause [e.g., contained connective tissue disease (CTD) related ILD] cannot be identified [4]. Although IIP and CTD-related ILD exhibit similar findings on computed tomography, their treatment response and prognosis differ [5]. Therefore, it is important to determine the presence or absence of CTD in patients with ILD to select appropriate treatment and predict prognosis. The Official American Thoracic Society/European Respiratory Society/Japanese Respiratory Society/Latin American Thoracic Association (ATS/ERS/JRS/ALAT) Clinical Practice Guideline recommends excluding CTD-related ILD by serological testing in the diagnosis of IIP [4]. Several reports revealed positivity for MSA in patients with IIP [2, 3]. However, there is no established protocol for examining the presence of MSA.

Previous reports have investigated the association between MSA and IIP [2, 3]. However, the prognostic value of MSA in patients with IIP is currently unknown. Therefore, we evaluated the effects of MSA, including its subtypes, on the incidence and mortality of acute exacerbation in patients with IIP.

## Methods

### Study design and participants

We retrospectively reviewed the medical records of all patients who visited the Department of Respiratory Medicine at Toho University Medical Center Sakura Hospital (Sakura, Japan) between December 2015 and July 2018. In our facility, all patients suspected of having ILD are evaluated for the presence of MSA. The current study included patients diagnosed with IIP in our institution. Patients diagnosed with idiopathic pulmonary fibrosis were included in IIP. Patients with IIP who met the following criteria were excluded from the analysis: (1) ILD caused by drugs, infection, autoimmune diseases (rheumatoid arthritis, scleroderma, dermatomyositis/polymyositis, Sjögren’s syndrome, antineutrophil cytoplasmic antibody-associated vasculitis, mixed CTD, systemic lupus erythematosus), or environmental exposure (radiation pneumonitis, asbestos lung); (2) other lung involvement besides ILD such as malignant tumor, emphysema, eosinophilic pneumonia, heart failure, hypersensitivity pneumonia; and (3) initial diagnosis of IIP, followed by diagnosis of IIM or CTD-related ILD.

Information regarding the date of death and onset date of acute exacerbation was obtained from the medical records of patients. Information was obtained for patients transferred to another medical institution by contacting the relevant facility. Acute exacerbation was defined following the Japanese diagnostic criteria described in the Hokkaido study [6]. The outcome was evaluated based on the status as of July 31, 2018. This study was approved by the Ethics Committee of Toho University Medical Center Sakura Hospital (approval number S20031) and conducted following the tenets of the Declaration of Helsinki and STROBE criteria of observational studies. Owing to this observational study’s retrospective nature, patient consent requirement was waived. Details about this study were disclosed on our website, and the potential participants were allowed to opt out.

### MSA

MSA was evaluated using the EUROLINE Myositis Antigens Profile 3 (IgG) Test kit (EUROIMMUN, Lubeck, Germany). This is a paper strip test that can evaluate 11 different antibodies (using the immunoblotting method) and seven subtypes of MSA (Jo-1 [antihistidyl], EJ [antiglycyl], OJ [anti-isoleucyl], PL-7 [antithreonyl], PL-12 [antialanyl], antisignal recognition particle [SRP], and antinucleosome remodeling complex [Mi-2, Mi-2α, Mi-2β]). Test results are interpreted based on color development through a catalase reaction, while the color intensity is evaluated using the signal intensity EUROLineScan flatbed scanner (EUROIMMUN). The color intensity is classified into five levels: (1) negative (−) 0–5; (2) borderline 6–10 (±); (3) positive 11–25 (+); (4) strong positive 26–50 (++); (5) very strong positive > 50 (+++). Levels 1–2 and 3–5 denoted negative and positive results, respectively. In addition, the enzyme-linked immunosorbent assay measured the levels of antibodies against malignant melanoma differentiation-associated protein 5 (MDA-5) and transcription intermediate factor-1γ (TIF-1γ). Survival time and duration from the first visit to acute exacerbation in patients with IIP were compared according to the subtype of MSA.

### Imaging

The ILD pattern was classified into two categories: acute course with symptoms (e.g., cough and dyspnea) within 1 month and chronic course with symptoms (e.g., cough and dyspnea) for > 1 month or without symptoms. Patients with an acute course were classified as having organizing pneumonia and diffuse alveolar damage according to the 2001 ATS/ERS International Multidisciplinary Consensus Classification of the Idiopathic Interstitial Pneumonias [7]. Patients with chronic disease were classified into usual interstitial pneumonia (UIP), possible UIP, or inconsistent with UIP according to the official ATS/ERS/JRS/ALAT statement: idiopathic pulmonary fibrosis 2009 [8]. Chest computed tomography images were evaluated (slice width: 1 mm) by at least two respiratory medicine specialists and a radiologist specializing in ILD to determine the ILD pattern.

### Treatments

The IIP treatments were categorized into the following classes: corticosteroids, immunosuppressants, and anti-fibrotic drugs. Corticosteroids included prednisolone, methylprednisolone, and dexamethasone; immunosuppressant drugs included cyclosporine, cyclophosphamide, and tacrolimus. Anti-fibrotic drugs included pirfenidone and nintedanib. Next, we compared acute exacerbations or deaths associated with each treatment class.

### Statistical analysis

Continuous variables are reported as the mean and standard deviation or median and interquartile range. P-values < 0.05 (two-tailed) indicate statistically significant differences. Predictors of survival time and duration from the first visit to acute exacerbation were assessed using Cox proportional hazards regression analysis. Univariate and multivariate models were constructed with a priori adjustments for sex, age, smoking history with Brinkman Index > 100, ILD pattern with UIP, and MSA (Jo-1, EJ, OJ, PL-7, PL-12, SRP, Mi-2). We adjusted the ILD pattern for UIP, which was linked to a poor prognosis by a previous study [8]. All statistical analyses were performed using the SPSS version 21.0 software (IBM Corp., Armonk, NY, USA).

## Results

**Fig. 1:**
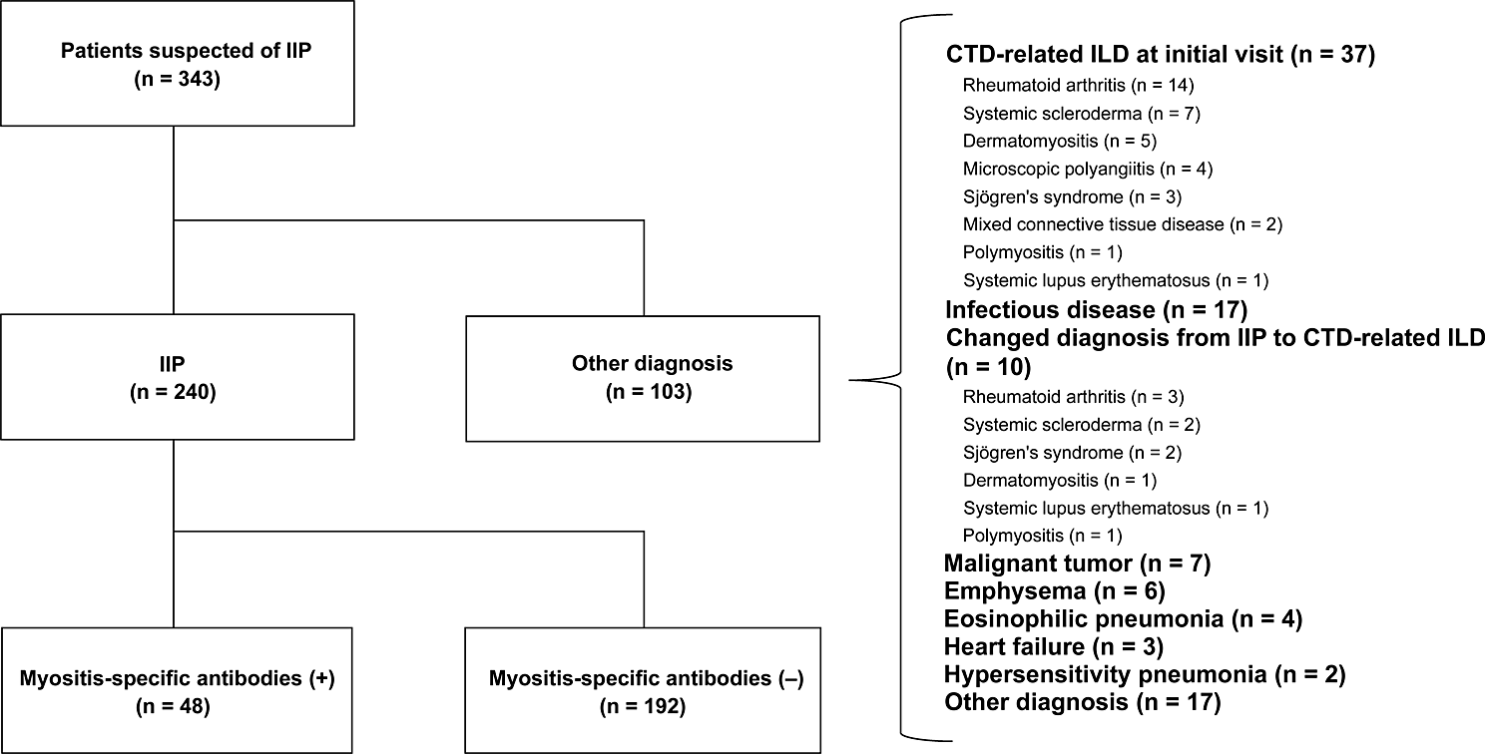
Study flowchart CTD, connective tissue disease; ILD, interstitial lung disease; IIP, idiopathic interstitial pneumonia

The study flow diagram is illustrated in Fig. 1. A total of 343 patients were suspected of having ILD; hence, they were evaluated for the presence of MSA. Of those, 240 patients (159 males [66%], 81 females [34%]; mean age: 71 ± 8.8 years) met the study inclusion criteria. Subjects were excluded due to CTD-related ILD at initial presentation (*n* = 37: rheumatoid arthritis [*n* = 14], systemic scleroderma [*n* = 7], dermatomyositis [*n* = 5], microscopic polyangiitis [*n* = 4], Sjögren’s syndrome [*n* = 3]), mixed CTD (*n* = 2), polymyositis [*n* = 1], systemic lupus erythematosus [*n* = 1]), infection in 17, changing diagnosis from IIP to CTD-related ILD (*n* = 10: rheumatoid arthritis [*n* = 3], systemic scleroderma [*n* = 2], Sjögren’s syndrome [*n* = 2], dermatomyositis [*n* = 1], polymyositis [*n* = 1], systemic lupus erythematosus [*n* = 1]), malignancy (*n* = 7), emphysema (*n* = 6), eosinophilic pneumonia (*n* = 4), heart failure (*n* = 2), hypersensitivity pneumonia (*n* = 1), and other diagnosis (*n* = 17). Table 1 provides information on the background, ILD pattern, outcome, and MSA profile of 240 patients with IIP. Notably, 58% of patients had a smoking history with Brinkman Index > 100. The most common ILD pattern was inconsistent with UIP (*n* = 80, 33%), followed by UIP (*n* = 65, 27%), possible UIP (*n* = 48, 20%), organizing pneumonia (*n* = 40, 17%), and diffuse alveolar damage (*n* = 7, 3%). During the 382 days (382 ± 281 days) of observation, acute exacerbation and death occurred in 27 (11%) and 32 (13%) patients, respectively. A total of 48 patients (20%) had MSA-related ILD. The most common MSA subtype was PL-7 (*n* = 16, 6.7%), followed by SRP (*n* = 13, 5.4%) and PL-12 (*n* = 9, 3.8%). Anti-ARS antibodies (PL-7, PL-12, OJ, EJ, and Jo-1) were detected in 75% of patients with MSA-related ILD. Only one patient (0.4%) exhibited positivity for Jo-1. All patients were negative for MDA-5 and TIF-1γ. Eight patients exhibited overlapping MSA positivities. Three were positive for PL-7 and PL-12, two had overlapping positivities for OJ and SRP, and one patient (each) had overlapping positivities for OJ and EJ, EJ and Mi-2, and OJ and PL-7.

**Table 1:** Characteristics of patients with idiopathic interstitial pneumonia

Characteristic	Number
Total patients	240
Sex	
Female	81 (34%)
Male	159 (66%)
Age at initial visit (years), mean ± SD	71 ± 8.8
Follow-up period (days), mean ± SD	382 ± 281
Smoking history (BI > 100)	139 (58%)
ILD pattern	
UIP	65 (27%)
Possible UIP	48 (20%)
Inconsistent with UIP	80 (33%)
Diffuse alveolar damage	7 (3%)
Organizing pneumonia	40 (17%)
Outcome	
Acute exacerbation	27 (11%)
Death	32 (13%)
Treatment	
Corticosteroids	96 (40%)
Immunosuppressant drugs	15 (6%)
Anti-fibrotic drugs	20 (8%)
MSA profile	
PL-7	16 (6.7%)
SRP	13 (5.4%)
PL-12	9 (3.8%)
Mi-2	8 (3.3%)
OJ	7 (2.9%)
EJ	3 (1.3%)
Jo-1	1 (0.4%)

BI, Brinkman Index; ILD, interstitial lung disease; MSA, myositis-specific antibodies; SD, standard deviation; UIP, usual interstitial pneumonia

**Fig. 2:**
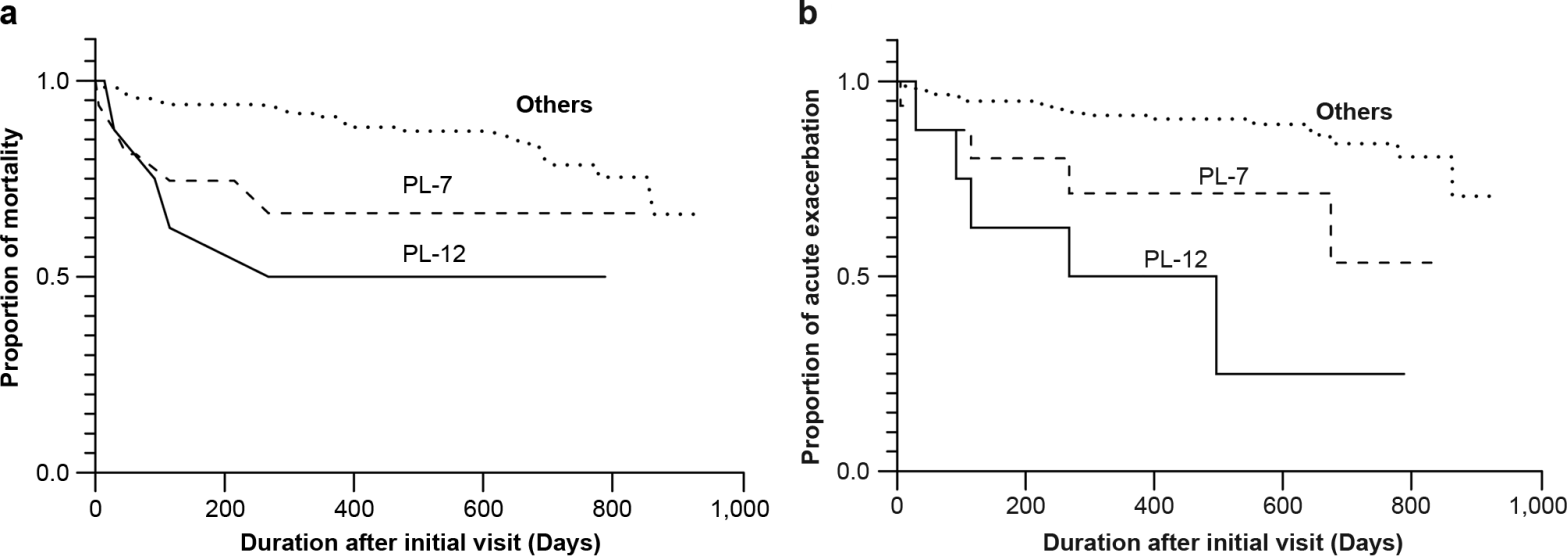
Kaplan–Meier curves for the estimation of mortality (a) and acute exacerbation (b) in PL-7-positive, PL-12-positive, and other patients with IIP. IIP; idiopathic interstitial pneumonias

Table 2 demonstrates the relationship between treatment and death or acute exacerbation. Of the 32 patients who died, 24 (75%) were treated with corticosteroids, 7 (29%) with immunosuppressants, and 4 (13%) with anti-fibrotic drugs. Both corticosteroids and immunosuppressant drugs were significantly associated with death (*P* < 0.001). All 26 patients who experienced acute exacerbations were treated with corticosteroids, 8 (31%) with immunosuppressants, and 1 (4%) with anti-fibrotic drugs. Treatment with either corticosteroids or immunosuppressants was significantly associated with an acute exacerbation (*P* < 0.001 for both). Cox proportional hazard regression models were used to examine mortality and acute exacerbation predictors in all patients with IIP during the follow-up. The model included sex and age at the initial visit, smoking history with Brinkman Index > 100, ILD pattern with UIP, and MSA (Jo-1, EJ, OJ, PL-7, PL-12, SRP, Mi-2). Cox proportional hazards regression (Table 3) and Kaplan–Meier curves (Fig. 2a) were used to estimate mortality. Age at initial visit (hazard ratio [HR]: 1.072; 95% confidence interval [CI]: 1.017–1.131; *P* = 0.01), PL-7 (HR: 4.785; 95% CI: 1.528–14.925; *P* = 0.007), and PL-12 (HR: 3.922; 95% CI: 1.198–12.821; *P* = 0.024) were identified as independent predictors of mortality.

**Table 2:** Correlation between treatment selection and patient outcomes

	Death (*n* = 32)	Survival (*n* = 208)		*P*-value
Corticosteroids	24 (75%)	72 (35%)		0.001 > *
Immunosuppressant drugs	7 (29%)	8 (4%)		0.001 > *
Anti-fibrotic drugs	4 (13%)	16 (8%)		0.318**
	Experienced Acute Exacerbation (*n* = 26)	Non-Experienced Acute Exacerbation (*n* = 214)		P-value
Corticosteroids	26 (100%)	70 (33%)		0.001 > *
Immunosuppressant drugs	8 (31%)	7 (3%)		0.001 > *
Anti-fibrotic drugs	1 (4%)	1 (0.5%)		0.705**

* Pearson chi-square test. ** Fisher’s exact test. Bold values are statistically significant (*P* < 0.05)

**Table 3:** Univariate and multivariate analyses of predictors of death for 240 patients with idiopathic interstitial pneumonia

	Univariate analysis			Multivariate analysis		
	Hazard ratio	95% confidence interval	P-value	Hazard ratio	95% confidence interval	P-value
Sex (male)	2.004	0.869–4.620	0.103	2.882	0.981–8.475	0.054
Age at initial visit	1.070	1.017–1.126	0.009	1.072	1.017–1.131	0.01
Smoking history (BI > 100)	1.080	0.537–2.171	0.830	0.604	0.239–1.529	0.287
ILD pattern (UIP)	1.593	0.797–3.188	0.188	2.049	0.921–4.566	0.079
Jo-1	0.049	0	0.875	0.000	0	0.995
EJ	0.048	0–41,337	0.664	0.000	0	0.982
OJ	2.466	0.588–10.335	0.217	1.919	0.402–9.174	0.414
PL-7	2.636	1.015–6.847	0.047	4.785	1.528–14.925	0.007
PL-12	4.435	1.552–12.675	0.005	3.922	1.198–12.821	0.024
SRP	1.389	0.332–5.821	0.653	1.425	0.314–6.452	0.647
Mi-2	0.763	0.104–5.591	0.790	1.082	0.141–8.264	0.939

BI, Brinkman Index; ILD, interstitial lung disease; UIP, usual interstitial pneumonia

Multivariate analysis was performed using models with variables selected a priori (sex and age at the initial visit, smoking history with Brinkman Index > 100), ILD pattern with UIP, MSA (Jo-1, EJ, OJ, PL-7, PL-12, SRP, and Mi-2). Bold values are statistically significant (*P* < 0.05)

The Cox proportional hazard regression (Table 4) and Kaplan–Meier curves (Fig. 2b) were also used to estimate acute exacerbation. PL-7 (HR: 3.268; 95% CI: 1.064–10; *P* = 0.039) and PL-12 (HR: 5.747; 95% CI: 1.894–7.544; *P* = 0.002) were recognized as independent predictors of acute exacerbation. Table 5 shows the ILD patterns, occurrence of acute exacerbation, and mortality in patients with IIP who were positive for PL-7 and PL-12 antibodies. There were 11, 6, and 3 patients with PL-7 single positivity, PL-12 single positivity, and overlapping positivity for PL-7 and PL-12, respectively. Of the 11 patients who exhibited PL-7 single positivity, 2 developed acute exacerbation, and 1 died. Of the six patients with PL-12 single positivity, two developed acute exacerbation, and one expired. All three patients who were positive for PL-7 and PL-12 developed acute exacerbation and expired.

**Table 4:** Univariate and multivariate analyses of predictors of acute exacerbation for 240 patients with idiopathic interstitial pneumonia

	Univariate analysis			Multivariate analysis		
	Hazard ratio	95% confidence interval	P-value	Hazard ratio	95% confidence interval	P-value
Sex (male)	1.277	0.559–2.918	0.562	1.597	0.529–4.831	0.407
Age at initial visit	1.046	0.992–1.102	0.100	1.052	0.996–1.112	0.069
Smoking history (BI > 100)	0.863	0.404–1.844	0.704	0.778	0.275–2.208	0.637
ILD pattern (UIP)	0.758	0.320–1.800	0.531	0.778	0.386–2.577	0.996
Jo-1	0.049	0	0.893	0.000	0	0.997
EJ	0.049	0–263,571	0702	0.000	0	0.99
OJ	1.551	0.210–11.471	0.667	1.321	0.158–10.989	0.797
PL-7	3.358	1.265–8.909	0.015	3.268	1.064–10	0.039
PL-12	7.532	2.824–20.088	0.001>	5.747	1.894–7.544	0.002
SRP	1.720	0.406–7.288	0.461	2.070	0.457–9.346	0.345
Mi-2	0.047	0–289	0.492	0.000	0	0.984

BI, Brinkman Index; ILD, interstitial lung disease; UIP, usual interstitial pneumonia

Multivariate analysis was performed using models with variables selected a priori (sex and age at the initial visit, smoking history with Brinkman Index > 100), ILD pattern with UIP, MSA (Jo-1, EJ, OJ, PL-7, PL-12, SRP, and Mi-2). Bold values are statistically significant (*P* < 0.05)

**Table 5:** Outcomes of patients with PL-7- and/or PL-12-positive idiopathic interstitial pneumonia

Patient no.	Sex	Age (years)	Follow-up period (days)	ILD pattern	Death	Acute exacerbation
PL-7 single positivity						
1	Female	68	536	OP		
2	Female	70	616	inc UIP	+	+
3	Male	44	794	inc UIP		
4	Female	71	5	inc UIP		
5	Female	61	834	inc UIP		
6	Female	79	194	p UIP		
7	Female	60	455	inc UIP		
8	Male	81	63	inc UIP		
9	Female	61	595	UIP		
10	Male	71	48	inc UIP		
11	Female	80	675	inc UIP	+	
PL-12 single positivity						
12	Male	83	443	p UIP		
13	Female	74	789	inc UIP		
14	Male	75	14	inc UIP		
15	Female	56	364	inc UIP		
16	Male	59	92	inc UIP	+	+
17	Male	67	497	DAD	+	
Overlapping positivity						
18	Male	72	268	inc UIP	+	+
19	Female	61	29	inc UIP	+	+
20	Male	68	115	inc UIP	+	+

DAD, diffuse alveolar damage; ILD, interstitial lung disease; OP, organizing pneumonia; UIP, usual interstitial pneumonia; inc UIP, inconsistent with UIP; p UIP, possible UIP. The (+) sign indicates patients who died or experienced an acute exacerbation

## Discussion

In this study, we investigated the presence and subtypes of MSA in patients with IIP and obtained two important findings. First, a significant number of patients with IIP were positive for MSA. Second, PL-7 and PL-12 were identified as independent risk factors for mortality and acute exacerbation in patients with IIP.

We found that 20% of patients with IIP exhibited positivity for MSA. Several reports have described the relationship between MSA and ILD. Fidler et al. screened patients with IIP for MSA and reported positive results in 11.4% [2]. Additionally, they reported that the diagnosis was changed in 8.5% of patients with IIP after screening for MSA. O’Mahony et al. investigated the presence of MSA in 165 patients with IIP; they identified 9.7% MSA-positive patients, and the diagnosis was changed in 6.0% of those [3]. The researchers described that screening patients with ILD for MSA is useful in reducing healthcare costs and the need for invasive examination of patients. MSA evaluations can be useful when diagnosing patients with ILD. Anti-ARS antibodies are among the MSA most commonly associated with ILD. They are divided into eight subgroups, namely Jo-1, PL-7, PL-12, OJ, EJ, KS, Zo, and Ha. Approximately 4–17% of patients with ILD exhibit positivity for anti-ARS antibodies [2, 3, 9, 10]. Jo-1 is the most common antibody in patients with IIM [1], detected in approximately 5% of patients with ILD [2, 9]. Patients with Jo-1-positive ILD prognosis [11] and those with Jo-1-positive anti-ARS syndrome have been linked to a good prognosis [12]. In our study of patients with IIP, 20% were positive for MSA; however, only 0.4% exhibited positivity for Jo-1. Although the positivity rate for MSA in patients with ILD was similar to that reported in previous studies [2], the positivity rate for Jo-1 was low. This observation may be due to targeting patients with IIP without any physical findings suggestive of myositis or autoimmune diseases. It has been reported that Jo-1 is present in 78–91% of patients with myositis [13] and is more frequently detected compared with other anti-ARS antibodies [10, 12]. Cases of Jo-1-positive IIP are rare, as Jo-1 is strongly associated with CTD-related ILD [14]. While the frequency of Raynaud’s phenomenon, arthritis, and rash is different depending on the study, anti-ARS antibodies other than Jo-1 have been reported to tend to develop interstitial pneumonia alone [15–18]. Furthermore, among patients meeting the criteria for interstitial pneumonia with autoimmune features (i.e., characteristics of individuals with ILD and features of CTD that do not satisfy the diagnostic criteria for CTD), PL-7, Mi-2, PL-12, and SRP are more commonly detected than Jo-1 [19]. This may explain the low detection rate of Jo-1 among anti-ARS antibodies in patients with IIP.

PL-7 and PL-12 were identified as independent risk factors for mortality and acute exacerbation in patients with IIP. The anti-ARS antibodies PL-7 and PL-12 are present in 5–10% of patients with IIM and dermatomyositis [15, 20]. Several reports described that PL-7 and PL-12 are strongly associated with ILD and disease behavior. In a study of 31 patients with positivity for PL-12, 28 (90%) had ILD; nevertheless, most patients were diagnosed with myositis or other autoimmune diseases, while only 3 patients were diagnosed with ILD alone [15]. In a Japanese study, most patients with PL-12 (89%) and PL-7 (76%) had ILD, while lower proportions of patients with PL-12 (33%) and PL-7 (14%) had ILD only. Moreover, PL-12-positive patients did not develop myositis during observation [21]. Prognostic differences between races have also been reported, with PL-12 and PL-7 implicated in more severe ILD in Blacks versus Whites [10]. Nevertheless, using multiple correspondence analysis in anti-ARS antibody-positive patients, Hervier et al. reported that multiple correlation analyses showing PL-7 and PL-12 are similar subtypes regarding ILD, no arthritis, no myositis, etc. In addition, multivariate Cox proportional hazards regression analysis indicated that PL-7 and PL-12 were not risk factors for mortality [22]. Zhan et al. also investigated 30 and 13 PL-7- and PL-12-positive patients among 108 anti-ARS antibody syndrome patients with ILD. They reported that > 90% of patients responded well to treatment [3]. These reports suggested that PL-7 and PL-12 are strongly associated with ILD, while the response to treatment and prognosis vary widely among the patients. The findings of our study suggested that PL-7 and PL-12 are strongly associated with ILD.

PL-7 and PL-12 were identified as independent risk factors for mortality; this finding differed from those of previous multivariate Cox proportional hazards regression analyses [22]. This difference may be attributed to several reasons, such as genetic, environmental, and race factors, as well as study design [10, 17].

Hervier et al. previously examined patients with autoimmune symptoms such as myositis, skin rashes, arthritis, or Raynaud’s phenomenon. They found that PL-7 and PL-12 were associated with severe lung involvement [22]. Our study was different in that we included asymptomatic (for suspected autoimmune issues) patients and found PL-7 and PL-12 to be independent risk factors for acute exacerbation and severe lung involvement. MDA-5 antibodies are another MSA subtype associated with severe lung involvement and poor prognosis. Patients with MDA-5-positive ILD are characterized by severe interstitial pneumonia without myositis” [23]. Therefore, we hypothesized that PL-7- and PL-12-positive patients without myositis would also develop severe ILD. Thus, PL-7 and PL-12 antibodies may predict poor prognosis in patients with IIP without symptoms indicative of autoimmune disease. This is the first report highlighting PL-7 and PL-12 as poor prognosis factors in patients with IIP.

In the present study, three patients exhibited overlapping positivity for PL-7 and PL-12; all three patients experienced acute exacerbation and expired. The co-occurrence of multiple anti-ARS antibodies is rare [17], and false-positive results may be obtained. However, as previously mentioned, PL-7 and PL-12 antibodies are similar antibodies and have been associated with severe lung involvement [22]. It would be interesting to examine whether patients who exhibit positivity for PL-7 and PL-12 have a worse prognosis than those who are positive for PL-7 or PL-12 alone. We hope that the number of cases exhibiting overlapping positivity for PL-7 and PL-12 will increase, and their characteristics will be determined in the future.

We found a significant association between treatment with corticosteroids or immunosuppressant drugs and acute exacerbations or death. This could be explained by acknowledging that corticosteroids are more likely to be prescribed to patients with severe lung involvement [8]. Immunosuppressant drugs are also used to treat patients with severe lung involvement or to reduce the corticosteroid dose [8]. We did not perform a multivariate analysis to examine potential associations between treatment and autoantibodies due to our small study cohort. However, a potential association between patients with MSA-positive ILD and treatment regimens is an intriguing area for future study.

This study has several limitations. First, this was a single-center study; thus, there may be recruitment bias. Second, considering the short follow-up period, patients with IIP may have developed myositis or anti-ARS antibody syndrome after the follow-up. Finally, it was a retrospective study based on electronic medical record data. Research involving prospective patient enrollment to evaluate the association of MSA with more accurate physical findings and subjective symptoms is warranted.

## Conclusions

In this study, we found that a significant number of patients with IIP also demonstrated MSA and that PL-7 and PL-12 were independent risk factors for mortality and acute exacerbation. The detection of MSA in patients with ILD may be useful in predicting prognosis and providing a rationale for intensive treatment.

## Data Availability

No datasets were generated or analysed during the current study.

## Abbreviations

MSA: myositis-specific antibodies
IIM: idiopathic inflammatory myopathy
ILD: interstitial lung disease
anti-ARS: anti-aminoacyl tRNA synthetase
IIP: idiopathic interstitial pneumonia
CTD: connective tissue disease
